# Genetic Diseases That Predispose to Early Liver Cirrhosis

**DOI:** 10.1155/2014/713754

**Published:** 2014-07-14

**Authors:** Manuela Scorza, Ausilia Elce, Federica Zarrilli, Renato Liguori, Felice Amato, Giuseppe Castaldo

**Affiliations:** ^1^CEINGE—Biotecnologie Avanzate Scarl, Via Gaetano Salvatore 486, 80145 Napoli, Italy; ^2^Dipartimento di Medicina Molecolare e Biotecnologie Mediche, Università di Napoli Federico II, Via Sergio Pansini 5, 80131 Napoli, Italy; ^3^Università Telematica Pegaso, Piazza Trieste e Trento 48, 80132 Napoli, Italy; ^4^Dipartimento di Bioscienze e Territorio, Università del Molise, Contrada Fonte Lappone, Pesche, 86090 Isernia, Italy

## Abstract

Inherited liver diseases are a group of metabolic and genetic defects that typically cause early chronic liver involvement. Most are due to a defect of an enzyme/transport protein that alters a metabolic pathway and exerts a pathogenic role mainly in the liver. The prevalence is variable, but most are rare pathologies. We review the pathophysiology of such diseases and the diagnostic contribution of laboratory tests, focusing on the role of molecular genetics. In fact, thanks to recent advances in genetics, molecular analysis permits early and specific diagnosis for most disorders and helps to reduce the invasive approach of liver biopsy.

## 1. Introduction

An early chronic liver involvement may be observed in a number of genetic and metabolic diseases although with different penetrance, age at onset, and outcome. Clinical symptoms and laboratory data are frequently overlapping, thus rendering a differential diagnosis difficult. A great improvement both in imaging [1] and in molecular genetics [2] in the last years helped to discriminate between the different diseases thus reducing the need of pathology (Table 1). On the other hand, liver biopsy is often complex in children, mainly due to the smaller specimen size [3]. For some diseases, prenatal diagnosis is also available [4].

Specific therapies are available for several genetic and metabolic diseases and their effectiveness is strongly related to the precocity of diagnosis. A growing number of children with such diseases now survive well into adulthood [5]. On the other hand, liver transplantation now offers a long-term survival [6].

We will review the genetic and metabolic entities responsible for early chronic liver diseases focusing on the contribution of laboratory and molecular diagnosis (Table 2).

## 2. Alpha-1 Antitrypsin Deficiency

Alpha-1 antitrypsin (AAT) deficiency (OMIM 613490) is an autosomal recessive (codominant) disease due to mutations in the* SERPINA1* gene that encodes the serine protease inhibitor AAT. The protein, mainly synthesized by liver cells, inhibits proinflammatory proteases such as neutrophil elastase, thus, protecting the lung from proteolytic damage. AAT deficiency has an incidence of 1 : 2,000–5,000 but the number of diagnosed patients is underestimated.

AAT deficiency appears with chronic obstructive pulmonary disease, emphysema, and disseminated bronchiectasis usually between the 4th and the 5th decade [7]. The liver involvement is widely heterogeneous according to age at onset (between the 1st year of life up to the 6th decade) and clinical severity that ranges from chronic hepatitis and cirrhosis to fulminant liver failure. The most likely pathogenic mechanism is the accumulation of AAT polymers in hepatocytes. The progression of liver disease is slow, even if few cases develop early cirrhosis with the need for transplantation; furthermore, hepatocellular carcinoma has a very high incidence among AAT deficient subjects [8]. The replacement therapy has no effect because liver damage is due to the accumulation of the AAT mutant polymers and not to the lack of circulating AAT [9].

The indications to laboratory diagnosis include various conditions exhaustively revised [10], but all infants with prolonged jaundice or nonspecific signs of liver disease should be tested for AAT deficiency.

Laboratory diagnosis includes serum AAT measured by nephelometry followed by the qualitative determination of AAT alleles by isoelectric focusing (IEF) and, finally, genotyping [11]. Serum AAT must be performed in subjects free from acute inflammation since AAT is an acute phase protein, and thus inflammation enhances its serum levels [12]. Then, AAT levels should not be considered if serum C-reactive protein levels are increased. The IEF analysis reveals the alleles of each subject. The M alleles (M1 to M6) are the most common and are considered “wild type alleles.” Most patients with liver or lung involvement are homozygous for the Z or the S or compound heterozygous for the two alleles. In these patients serum AAT levels are reduced by 40–60%. Heterozygous individuals (MZ or MS) rarely develop clinical signs. However, the IEF analysis may provide false negative results. Also for this reason, molecular analysis is indicated [11]. In the* SERPINA1* disease gene (official name: serpin peptidase inhibitor, clade A), more than 120 allelic variants have been identified so far; thus molecular analysis must be performed by gene sequencing. Gene variants are classified according to their effect on serum levels of alpha-1 antitrypsin [13]. Patients must be counseled before molecular analysis, and when severe mutations are identified the consanguineous should be counseled in turn and analyzed to reveal asymptomatic carriers.

## 3. Cystic Fibrosis

Cystic fibrosis (CF, OMIM 219700) is the most frequent lethal autosomal recessive disease among Caucasians (incidence: 1 : 2,500). CF is a systemic disease that appears mainly with pancreatic insufficiency in more than 90% of cases and pulmonary disease due to inflammation and opportunistic colonization that gradually causes respiratory insufficiency [14]. About 20% of patients experience meconium ileus.

Liver disease in CF appears mainly in the first decade of life and it is observed in up to 30% of patients, but it is still obscure why only some patients develop liver disease. In fact, CF liver disease depends on the altered activity of cystic fibrosis transmembrane regulator (CFTR) chloride channel on the apical membrane of cholangiocytes. It causes an altered bile flow followed by a cholangiocyte-induced inflammatory response with proliferation of stellate cells, which gives rise to cholangitis and progressive periportal fibrosis [15]. CF-associated liver disease is slowly progressive, but in up to 10% of patients it may rapidly evolve to multilobular biliary cirrhosis and portal hypertension.

The possibility to predict CF patients that will develop a severe liver involvement would be useful given the efficacy of ursodeoxycholic acid in the early stages of liver disease [15], but its pathogenesis is poorly known. The scarce correlation between the* CFTR* genotype and liver expression in CF patients is well known [16], as it is the discordant severity of liver disease in CF sib pairs [17]. A decade of studies concluded that liver expression in CF patients is influenced by modifier genes like mannose-binding lectin and AAT [18], but such genes modulate the risk for liver disease only in a small percentage of CF patients.

Finally, clinical forms showing pancreatic sufficiency, single organ involvement, and a milder outcome are included under the term of* CFTR*-related disorders (*CFTR*-RD) [19]. These disorders are not associated with liver disease.

The gold standard for CF diagnosis is the sweat test (i.e., sweat chloride levels after pilocarpine stimulation) followed by molecular analysis. Sweat test requires a specific skill and the knowledge of all conditions that may cause false positives, while the rate of false negative results is very low. However, CF diagnosis or exclusion must be based on two concordant sweat tests. The indications to sweat test include a large variety of clinical conditions [14, 15].

The search of* CFTR* mutations is one of the most diffuse molecular analyses worldwide, because it is used to confirm CF diagnosis and prenatal [20] or preimplantation [21] diagnosis. About 2000 mutations have been identified in the* CFTR* gene so far (http://www.genet.sickkids.on.ca/). Guidelines suggest a two-step molecular analysis. In the first step a panel of the most frequent mutations is analyzed, including the mutations peculiar to the geographic area of each patient [22], and commercial kits are used [16, 23]. The first step identifies about 80% alleles from CF patients; the analysis of mutations peculiar to specific ethnic or geographic groups may increase the detection rate [24], and the scanning of* CFTR* coding regions reveals mutations in up to 90% alleles [25]. Large gene rearrangements are present in about 2-3% of CF alleles [16]. Finally, pathogenic mutations in noncoding region of the* CFTR* gene have been described [26, 27], but they are not analyzed for routine purposes. The detection rate of molecular analysis is lower in* CFTR*-RD [28]. No mutations are specifically associated with liver disease [29].

The diagnosis of liver disease in CF patients is difficult, because neither laboratory nor imaging has a great specificity. Liver biopsy contributes to the aim, but the patchy distribution of liver alterations in CF patients reduces its sensitivity [15].

## 4. Wilson Disease

Wilson disease (WD, 277900) is an autosomal recessive disorder with an incidence of about 1 : 30,000. It typically appears with liver disease in the second decade and neurological disorders in the third decade, even if cases with earlier or later onset have been described [30]. Wilson disease depends on mutations in the gene encoding the ATP7B Cu translocase, a protein mainly expressed by the hepatocyte that regulates the levels of copper in the liver. Furthermore, ATP7B modulates the synthesis of ceruloplasmin [31]. When the activity of ATP7B is reduced, copper accumulates within the hepatocyte. The severity of liver involvement in WD patients is heterogeneous ranging from asymptomatic cases with mild hepatomegaly to cirrhosis with severe liver failure [32]. About two-thirds of patients show haemolytic anemia, coagulopathy, and renal failure. About 6% of WD cases appear with acute liver onset [33]. Kayser-Fleischer rings are present in about 50% of WD cases at diagnosis. About half of patients show psychiatric alterations, up to psychotic symptoms, reverted by adequate therapy [34]. Novel therapies can now effectively treat WD patients and gene therapy is effective in animal models [35].

Low serum ceruloplasmin and high urine copper help to perform the diagnosis in most cases, even if some false negatives are described [35].

Molecular analysis is available [36, 37]. Wilson disease is due to mutations in the* ATP7B* gene: about 300 different mutations are known so far (http://www.wilsondisease.med.ualberta.ca/database.asp); thus gene sequencing is required, reaching a detection rate of about 95%. Severe mutations (i.e., nonsense, frameshift) are associated with the most severe disease, while patients with missense mutations (about 60% of all mutations) show a variable severity and outcome [30]. Liver biopsy is now used in cases with ambiguous biochemical parameters and to evaluate liver copper levels [3].

## 5. Hereditary Hemochromatosis

Hereditary hemochromatosis (HH, OMIM 235200) is an autosomal recessive disease characterized by iron overload that may cause liver cirrhosis, cardiomyopathy, diabetes, arthritis, and skin pigmentation that appear during the third to fifth decade. The incidence is about 1 : 250. A myriad of diseases cause secondary hemochromatosis. However, while in secondary hemochromatosis the iron overload involves macrophages; in HH iron mainly accumulates in hepatocytes, causing chronic liver damage that ends in cirrhosis [38], with a percentage of cases evolving to hepatocellular carcinoma [39]. The pathogenesis of liver damage in HH is mainly due to the iron-induced lipid peroxidation that occurs in hepatocytes and causes hepatocellular injury or death. Kupffer cells become activated and produce cytokines, which in turn stimulate hepatic stellate cells to synthesize collagen, thereby leading to cirrhosis [38]. Symptoms of hemochromatosis depend on the phase of the disease. When HH is diagnosed by occasional laboratory evaluation, most patients are still asymptomatic; if the diagnosis is performed for symptoms, HH may appear with cirrhosis, bronze-colored skin, diabetes (and other endocrine diseases), joint inflammation, heart disease, arthralgia, and hepatomegaly.

The diagnosis is based on enhanced serum ferritin that correlates with the increased iron content of liver and the high transferrin saturation. Unsaturated iron-binding capacity has been proposed as an alternative to transferrin saturation [40], but it has a higher biological variability [41]. Molecular analysis in* HFE* gene with different protocols [42–47] would confirm hereditary hemochromatosis but surprisingly it has a lower diagnostic sensitivity because the mutations are different in each geographic area. Homozygous patients for p.Cys282Tyr have a higher risk for iron overload.

Liver biopsy is performed in suspected patients with negative molecular analysis and ambiguous laboratory results; in addition, it may be used to assess the degree of liver fibrosis and cirrhosis and the degree of iron liver overload [3, 48]. Laboratory has a role also in the monitoring of patients through biochemical markers of (i) liver fibrosis [49]; (ii) liver protidosynthesis [50]; and (iii) hepatocarcinoma in patients with cirrhosis [51].

## 6. Type I Tyrosinemia

Type I tyrosinemia (TYRSN1, OMIM 276700) is an autosomal recessive disease with an incidence of about 1 : 100,000. It is the most severe form of genetic tyrosinemia and is the only one that causes a severe liver involvement [52, 53].

Type I tyrosinemia is classified in two forms: the first, more frequent, appears with a severe liver expression in the first months of life that may progress to ascites, jaundice, and gastrointestinal bleeding; the second includes cases with acute liver failure at about one year and a chronic evolution with renal-tubular dysfunction [54]. Untreated patients die within the first decade of liver failure or of early hepatocarcinoma. The use of nitisinone and a tyrosine-restricted diet quite completely revert symptoms [55]. Type I tyrosinemia is due to the altered activity of fumarylacetoacetate hydrolase, which causes the elevation of plasma and urine succinylacetone (diagnostic golden standard) and high plasma concentration of tyrosine, methionine, and phenylalanine. Sequence analysis in* FAH* gene may be performed for molecular diagnosis including prenatal [56].

## 7. Glycogen Storage Disease Type IV

Glycogen storage disease (GSD, OMIM 232500) type IV is an autosomal recessive disease with an incidence of about 1 : 600,000. It is due to mutations in the gene encoding the glycogen branching enzyme (GBE1) that catalyzes the alpha 1,6 bond of the first glucose in the side chains of glycogen [57]. The altered glycogen branching reduces its solubility, thus impairing the osmotic pressure within the hepatocyte [58]. Several clinical forms of GSD have been described including (i) a neuromuscular form that appears in the perinatal age or in childhood; most of these cases have an early fatal evolution and are typically due to two null mutations; (ii) a hepatic form that may have a progressive or a nonprogressive evolution; patients are usually compound heterozygous for a severe (null) and a mild (missense) mutation; and (iii) the polyglucosan body disease that appears in adulthood with upper and lower motor neuron involvement and executive dysfunction [59]. The hepatic form is the most frequent phenotype. In the progressive subtype, the clinical expression appears in the first months of life with failure to thrive and hepatomegaly that evolves (in a variable time) to cirrhosis with portal hypertension requiring liver transplantation [60]. In the rare nonprogressive subtype the patients show a variable combination of liver disease (that usually does not evolve to cirrhosis), myopathy, and hypotonia. The diagnosis of GSD is based on biochemical findings from a liver biopsy that reveals an abnormal glycogen content, and on the evidence of enzymatic deficiency in the liver, muscle, or fibroblasts. Now it is based on molecular analysis, that is, the sequence of the* GBE1* gene followed by the search of large gene deletions [61]. In about 10% of patients a negative molecular analysis despite clinical symptoms suggests to perform the enzyme assay on cultured fibroblasts [62]. Prenatal diagnosis is possible if the disease-causing mutations in the family proband are known [63].

## 8. The Urea Cycle

The urea cycle includes a series of reactions that convert nitrogen from ammonia and aspartate into urea [64]. Urea cycle disorders (UCDs) are a group of inborn errors that typically cause a life-threatening hyperammonemia. Among these, argininosuccinate lyase (ASL) and citrin deficiency are usually associated with severe liver disease.

### 8.1. Argininosuccinate Lyase Deficiency

Argininosuccinate lyase deficiency (ASLD, OMIM 207900) is the second most common UCD with an incidence of about 1 : 70,000 and is due to the deficiency of the enzyme that cleaves argininosuccinic acid into arginine and fumarate. The disease includes a severe neonatal onset form and a late onset form: the first appears with hyperammonemia within the first few days of life with vomiting, lethargy, hypothermia, and poor feeding. On the contrary, the late onset form ranges from episodic hyperammonemia triggered by acute infections or stress to cognitive impairment, behavioral abnormalities, and/or learning disabilities in the absence of episodes of hyperammonemia [65]. Symptoms of ASL deficiency are unrelated to the severity or duration of hyperammonemic episodes and include neurocognitive deficiencies with attention deficit hyperactivity disorder and developmental disability [66]. Liver disease ranges from hepatomegaly to severe liver fibrosis and cirrhosis [67]. Systemic hypertension [68] and electrolyte imbalance may be present.

Laboratory diagnosis of ASL deficiency is based on enhanced levels of citrulline and increased argininosuccinic acid in plasma and/or in urine [64]. A newborn screening for ASLD is available in all US citrulline testing. ASLD is due to heterogeneous mutations in the* ASL* gene [69] and sequence analysis detects mutations in about 90% of patients. ASL enzyme activity can be measured in cell homogenates from liver biopsy or from skin fibroblasts or erythrocytes [70]. Prenatal diagnosis is available [71].

### 8.2. Citrin Deficiency

Citrin deficiency is an autosomal recessive disorder and may appear with two phenotypes: neonatal intrahepatic cholestasis caused by citrin deficiency (NICCD, OMIM 605814) and the adult form called citrullinemia type II (CTLN_2_, OMIM 603471). A form that appears with dyslipidemia (FTTDCD) was described more recently [72]. Typically, citrin deficiency is characterized by food preference (protein-rich/lipid-rich foods) or aversion (carbohydrate-rich foods).

Neonatal intrahepatic cholestasis has an incidence of about 1 : 19,000 and appears with aminoacidemias, galactosemia, hypoproteinemia, cholestasis, and variable hepatic dysfunction [73]. Although such symptoms self-resolve by the first year in most cases, some infants die of infection or of liver cirrhosis [74, 75]. Citrullinemia type II has an incidence of about 1 : 100,000 and appears later between second and fourth decade with recurrent hyperammonemia with neuropsychiatric symptoms; death can be due to brain edema [76]. Symptoms are frequently triggered by alcohol and sugar intake, medication, and/or surgery. Affected patients may or may not have a prior history of NICCD or FTTDCD.

The diagnosis of citrin deficiency is based on clinical and biochemical findings that include enhanced plasma ammonia, citrulline and arginine, and threonine : serine ratio. In neonatal intrahepatic cholestasis plasma threonine, methionine, and tyrosine are elevated as bilirubin, bile acids, and alpha-fetoprotein [73]. Plasma levels of galactose, methionine, and/or phenylalanine are elevated in newborn screening blood spots in about 40% of children.

Citrin deficiency is caused by mutations in* SLC25A13* gene and is characterized by a liver-specific decrease in argininosuccinate synthetase (ASS) [77, 78]. The liver reduction of ASS in CTLN2 patients is secondary to citrin deficiency [79], although its cause still remains to be clarified.

## 9. Cholesteryl Ester Storage Disease

Cholesterol ester storage disease (CESD, OMIM 278000) is an autosomal recessive disorder of lysosomal storage with an incidence ranging between 1 in 40,000 in the Germanic population and 1 : 300,000–1 : 500,000 in the general Caucasian population [80]. It is due to deficiency of lysosomal acid lipase (LAL), which catalyzes the intracellular hydrolysis of triacylglycerols and cholesteryl esters. Its deficiency causes a progressive accumulation of cholesteryl esters (CE), and to a lesser extent, triglycerides, mainly in lysosomal hepatocytes, adrenal glands, and macrophages [81].

Usually patients develop hepatomegaly that leads to fibrosis and micronodular cirrhosis [82] within the first ten years of life. CESD can appear as two forms: Wolman disease, that is, the severe pediatric form, fatal within 1-2 years of life, and the later onset CESD, a more benign disease, associated with some residual LAL activity [80].

Wolman disease is a rare, neonatal onset, lethal disorder that appears in the first months of life with vomiting and diarrhea and severe hepatosplenomegaly. About 50% of patients show adrenal calcifications [83]. In contrast, CESD is often undiagnosed, has a later onset, and may appear in infancy or childhood, depending on the residual levels of LAL activity [83, 84]. CESD should be suspected in children with hepatomegaly and splenomegaly with elevated transaminases, high cholesterol, and low HDL [85].

Liver biopsy helps the diagnosis even if false negatives were reported [86, 87]. To confirm the diagnosis of CESD, LAL activity and molecular analysis of the acid lipase gene (*LIPA*) are available.

To date, over 40* LIPA* mutations have been identified in patients with CESD [88]. No genotype-phenotype correlation has been established. Prenatal diagnosis is also available [80].

## 10. Alström Syndrome

Alström syndrome (ALMS, OMIM 203800) is a rare autosomal recessive disease with an incidence of 1 : 1,000,000. It appears in infancy with a wide variability in age at onset and severity, and typically leads to organ failure causing a reduced life expectancy, rarely exceeding 50 years. Alström syndrome appears with cone-rod dystrophy, obesity, progressive sensorineural hearing impairment, dilated or restrictive cardiomyopathy, the insulin resistance syndrome, and multiple organ failure [89, 90]. Therapy is complex due to the combination of multiple endocrine disorders, sensorineural deficits, cardiac, renal, and hepatic abnormalities [91]. Fibrosis develops in multiple organs [89]. Liver expression ranges from steatohepatitis to portal hypertension and cirrhosis and can cause hepatic encephalopathy and life-threatening esophageal varices. The diagnosis is based on clinical features [92], and genetic testing is used when major (vision) and minor criteria do not permit a clinical diagnosis. Molecular testing of the disease gene,* ALMS1, *detects mutations in up to 80% of patients of northern European descent, and in about 40% of cases worldwide [93, 94]. Carrier and prenatal diagnosis can be offered if the disease-causing mutations have been identified in a family proband [95].

## 11. Congenital Hepatic Fibrosis

Congenital hepatic fibrosis (CHF) is an autosomal recessive disease characterized by periportal fibrosis and irregularly shaped proliferating bile ducts. The incidence is about 1 : 20,000 [96].

In most patients, the first symptom is portal hypertension (PH) with gastrointestinal bleeding [97]. Pulmonary hypertension and pulmonary vascular shunts are typical complications of PH. Frequently CHF is associated with ciliopathies and renal disease, the so-called hepatorenal fibrocystic disease [98].

Congenital hepatic fibrosis involves various organs (e.g. renal, central nervous system, etc.), but most cases are referred for liver diseases. Four clinical forms have been described [99]: (i) portal hypertension (most common and more severe in the presence of portal vein abnormality); (ii) cholangitis with cholestasis and recurrent cholangitis; (iii) both portal hypertension and cholangitic symptoms; and (iv) latency that appears at a late age with hard hepatomegaly.

Symptoms of CHF are nonspecific, making the diagnosis difficult. The late onset and the clinical evolution suggest that CHF is a dynamic and progressive condition [100, 101].

The diagnosis of CHF can be made by liver biopsy that shows a progressive hepatic fibrosis with nodular formation. Such findings may be mistaken for cirrhosis, but, unlike cirrhosis, hepatic lobules are usually normal with normal hepatocyte morphology, particularly in the early phases [100, 102]. The gene/s causing CHF is/are unknown.

## 12. Hereditary Fructose Intolerance

Hereditary fructose intolerance (HFI, OMIM 229600) is an autosomal recessive disease (incidence 1 : 20,000) due to the deficiency of fructose 1-phosphate aldolase (aldolase B) involved in the metabolism of fructose-1-phosphate (exogenous fructose) into dihydroxyacetone phosphate and D-glyceraldehyde [103].

Onset of symptoms can occur at any age. The persistent intake of fructose, sucrose, or sorbitol in childhood leads to chronic toxicity [104, 105] that causes irreversible damage to the liver (early cirrhosis) and kidney [105]. The strict dietary exclusion leads to normal growth and longevity, but it is difficult to achieve [106].

The early diagnosis of HFI is crucial to start the strict exclusion diet thus avoiding tissue injury and growthretardation. ^31^P nuclear magnetic resonance spectroscopy has been used successfully [107]. The fructose tolerance test (breath test) has a high diagnostic sensitivity [106]. Molecular diagnosis of HFI consists in direct sequencing of the gene encoding aldolase B (*ALDOB*). About 45 different mutations are known so far (http://www.bu.edu/aldolase/HFI/hfidb/hfidb.html) and it is now the diagnostic gold standard [108–111].

## 13. Progressive Familial Intrahepatic Cholestasis Type 3

Progressive familial intrahepatic cholestasis (PFIC) refers to a heterogeneous group of inherited cholestatic disorders that impair bile formation and appear with cholestasis of hepatocellular origin. Three types of PFIC are known. Progressive familial intrahepatic cholestasis type 3 (PFIC3, OMIM 602347) is an autosomal recessive disorder with a prevalence estimated of about 1 : 100,000 [112].

PFIC3 may appear in infancy, in childhood, or during young adulthood. Main symptoms include gastrointestinal bleeding due to portal hypertension, early cirrhosis, and moderate pruritus [113]. The phenotypic expression of PFIC3 ranges from neonatal cholestasis to cirrhosis in young adults [114]. The evolution of the disease is characterized by chronic icteric or anicteric cholestasis, portal hypertension, and liver failure. In about 50% of the patients, liver transplantation is required at a mean age of 7.5 years [115]. Laboratory findings show high serum gamma-glutamyl transferase (*γ*-GT) activity (while other two types of PFIC have normal serum *γ*-GT activity), normal cholesterol levels, and moderately enhanced bile acid concentrations. Liver histology shows portal fibrosis and true ductular proliferation with mixed inflammatory infiltrate and, in advanced phases, signs of biliary cirrhosis. Interlobular bile ducts are seen in most portal tracts and there is neither periductal fibrosis nor biliary epithelium injury [113].

PFIC3 is caused by mutations in the* ABCB4 *gene encoding the multidrug resistance protein 3 (MDR3) protein. This gene is expressed in the canalicular membrane of the hepatocyte and is responsible for phospholipid transport into bile [116]. Reduced or absent activity of the MDR3 transporter causes impaired phospholipid secretion, previously identified as “low phospholipid syndrome” [117]. The diagnosis of PFIC3 is confirmed by molecular genetic analysis of the* ABCB4 *gene by sequencing of exons and their splice junctions (http://evs.gs.washington.edu/EVS/) [118]. There are several mutations in* ABCB4 *that have a clear effect on the protein and a genotype-phenotype correlation is observed [119–122]. Prenatal diagnosis is available.

## 14. Conclusions and Future Prospects

A chronic liver involvement that can predispose to cirrhosis may be observed in a number of genetic diseases with a different penetrance, age at onset, and outcome. Clinical symptoms and laboratory data are frequently overlapping, thus rendering a differential diagnosis difficult. In the present review we critically discussed the genetic entities responsible for early liver cirrhosis, describing for each disease the laboratory diagnosis and molecular genetics.

In fact, the recent advances made in understanding the genetics and pathophysiology of inherited liver diseases can contribute to the identification of novel strategies for the diagnosis of these conditions. Molecular analysis changed the diagnostic approach in these genetic diseases and led to reduction of invasive and expensive procedures and diagnostic errors.

Disease-genes identification is a step forward in the diagnostic approach to a patient in whom early liver cirrhosisis strongly suspected. However, we have to point out some critical points: (i) molecular analysis would be based on scanning procedures using the gene sequencing; (ii) the negative result of molecular analysis does not exclude the disease, because mutations may involve noncoding, regulatory areas; (iii) some liver diseases are very rare; it is necessary that laboratories also offer molecular diagnosis for such diseases.

However, the availability of new technologies as high throughput sequencing at reasonable costs could help to perform extensive analyses, especially in cases in which more disease genes are involved. No clear genotype-phenotype correlation has been established in most cases, so proteomic and functional studies on the effect of the mutations may guide physicians in the prescription of treatment procedures. In some cases, molecular analysis has been used for prenatal diagnosis to help high risk couples to better plan their reproductive options.

However, given the increased number of genetic liver diseases, the complexity of genotype-phenotype correlations and the need of multidisciplinary counseling to the families, a strict collaboration between physicians and molecular laboratories is mandatory in this field.

## Figures and Tables

**Table 1:** Inherited liver diseases that predispose to early cirrhosis.

Disease	Incidence	Gene
Disorders of bile acid synthesis		
Wilson disease	1 : 30,000	*ATP7B *
Progressive familial intrahepatic cholestasis type 3	1 : 100,000	*ABCB4 *
Disorders of carbohydrate metabolism		
Hereditary fructose intolerance	1 : 20,000	*ALDOB *
Glycogen storage disease type IV	1 : 600,000	*GBE1 *
Disorders of amino acids metabolism		
Tyrosinemia type I	1 : 100,000	*FAH *
Urea cycle disorders		
Argininosuccinate lyase deficiency	1 : 70,000	*ASL *
Citrin deficiency (CTLN2, NICCD)	CTLN2 1 : 100,000 NICCD 1 : 19,000	*SLC25A13 *
Disorders of lipid metabolism		
Cholesteryl ester storage disease	1 : 40,000 (Germany) 1 : 300,000–1 : 500,000	*LIPA *
Other diseases		
Alpha-1 antitrypsin deficiency	1 : 2,000–1 : 5,000	*SERPINA1 *
Cystic fibrosis	1 : 2,500	*CFTR *
Hereditary hemochromatosis	1 : 250	*HFE *
Alström syndrome	1 : 1.000.000	*ALMS1 *
Congenital hepatic fibrosis	1 : 20,000	Unknown

**Table 2:** Main characteristics of genetic liver disease that predispose to early cirrhosis.

	Age at onset (ys)	Pathogenic mechanism of liver damage	Laboratory diagnosis	Molecular genetics
Alpha-1 antitrypsin deficiency	40–50	Accumulation of AAT polymers in hepatocytes	Low serum AAT; AAT alleles by isoelectric focusing	120 allelic variants in *SERPINA1 *gene; ZZ genotype associated with liver cirrhosis

Cystic fibrosis	0–12	Altered activity of CFTR; increased bile flow that causes cholangitis and fibrosis	Sweat test	About 2000 known mutations in *CFTR* gene; no mutation specific for liver disease

Wilson disease	20–22	Copper hepatocyte increased levels dislocate the ATP7B protein impairing copper excretion through the bile	Low serum ceruloplasmin; high urine copper	About 300 known mutations in *ATP7B *gene; severe mutations (nonsense, frameshift) are associated with liver disease

Hereditary hemochromatosis	30–50	Iron-induced lipid peroxidation causes hepatocellular injury	Enhanced serum ferritin; high transferrin saturation	p.C282Y in *HFE* gene associated with liver cirrhosis

Type I tyrosinemia	Variable	The metabolite succinylacetone accumulates, resulting in toxicity to liver	Enhanced plasma/urine succinylacetone; high plasma tyrosine, methionine, and phenylalanine	Most frequent mutations analysis in *FAH* gene; no mutation specific for liver disease

Glycogen storage disease type IV	Variable	The altered stored glycogen impairs the osmotic pressure within the hepatocyte	/	Sequence analysis in *GBE1 *gene*; *no mutation specific for liver disease

Argininosuccinate lyase deficiency	0–15	Decreased endogenous synthesis of arginine that leads to a decrease in arginine metabolites in various tissues	High serum citrulline; increased argininosuccinic acid in plasma/urine	*ASL *exons 4, 5, and 7 are hotspots of most frequent mutations; no mutation specific for liver disease

Citrin deficiency	NICCD: 0-1; CTLN2: 20–40	Defective aspartate export from the mitochondria to the cytosol and defects in the malate aspartate shuttle	Enhanced plasma ammonia, citrulline, and arginine. NICCD: high plasma threonine, methionine, tyrosine, bilirubin, and bile acids	Sequence analysis in *SLC25A13* gene*; *no mutation specific for liver disease

Cholesteryl ester storage disease	0–20	Accumulation of cholesteryl esters and triglycerides in lysosomal hepatocytes	High serum AST, ALT, cholesterol, and low HDL cholesterol	About 40 mutations in *LIPA* gene; (exons 16, 10, and 8 are hotspots of mutations); no mutation specific for liver disease

Alström syndrome	Variable	Unclear mechanism	/	About 80 mutations in *ALMS1* gene; no mutation specific for liver disease

Congenital hepatic fibrosis	Variable	Immature duct structures stimulate the formation of portal fibrous tissue	/	The disease gene is unknown

Hereditary fructose intolerance	Variable	Accumulation of fructose in hepatocytes; fibrosis	Breath test	About 45 known mutations in *ALDOB *gene; no mutation specific for liver disease

Progressive familial intrahepatic cholestasis type 3	0–20	The defect of MDR3 results in impaired biliary phospholipid excretion that impairs bile formation	High serum *γ*-GT activity, normal serum cholesterol and moderately raised bile salts concentrations	Most of known mutations in *ABCB4* are point mutations
